# Isolation of *Beauveria* Strains and Their Potential as Control Agents for *Lema bilineata* Germar (Coleoptera: Chrysomelidae)

**DOI:** 10.3390/insects13010093

**Published:** 2022-01-14

**Authors:** Jason Lee Furuie, Andressa Katiski da Costa Stuart, Morgana Ferreira Voidaleski, Maria Aparecida Cassilha Zawadneak, Ida Chapaval Pimentel

**Affiliations:** 1 Laboratory of Microbiology and Molecular Biology (LabMicro), Department of Basic Pathology, Federal University of Paraná, Av. Cel. Francisco H. dos Santos, 100, Curitiba CEP 81530-900, Brazil; andressa.katiski@gmail.com (A.K.d.C.S.); morganavoidaleski@gmail.com (M.F.V.); ida@ufpr.br (I.C.P.); 2 Laboratory of Entomology Professor Ângelo Moreira da Costa Lima, Department of Basic Pathology, Federal University of Paraná, Av. Cel. Francisco H. dos Santos, 100, Curitiba CEP 81530-900, Brazil; mazawa@ufpr.br

**Keywords:** pathogenicity, entomopathogenic fungi, integrated pest management, *Physalis peruviana*

## Abstract

**Simple Summary:**

The use of fungi as biocontrol agents against insect pests can be an environmentally friendly alternative to the use of chemical pesticides. In this study, 15 fungal strains were isolated and identified as *Beauveria bassiana*, which is a fungus that can harm insects. Consequently, we tested these strains, under laboratory conditions, against adults of *Lema bilineata* Germar (Coleoptera: Chrysomelidae), which is an emerging pest in Brazil. These insects showed a great sensitivity to three of the fungal isolates tested. Then, these three fungal strains were applied to the eggs of this insect and showed a rather high ovicidal capacity. Our findings showed that these fungal isolates, which have pathogenic potential as biocontrol agents against *L. bilineata*, are a promising alternative to chemical insecticides. However, these results must be expanded with experiments in open fields.

**Abstract:**

*Lema bilineata* Germar (Coleoptera: Chrysomelidae) was recently reported to damage *Physalis peruviana* crops in Brazil. Given the potential for inflicting damage on other Solanaceae species and the lack of alternatives for controlling this pest, we assessed the pathogenicity of 15 *Beauveria* isolates against *L. bilineata* adults in vitro. In addition, three of these isolates were tested for their ovicidal effect against *L. bilineata* eggs. Fungal strains were isolated from mummified corpses of *L. bilineata* collected in a non-commercial field in Paraná, Brazil. The isolates were identified as *Beauveria bassiana* using molecular markers. *Lema bilineata* adults were susceptible to conidial suspensions of all these isolates at a concentration of 10^8^ conidia mL^−1^. Deaths caused by fungal extrusion were confirmed. Three strains were found to be more virulent against *L. bilineata* adults and showed ovicidal effects. This is the first study on entomopathogenic fungi isolated from dead insects collected from *P. peruviana* crops and tested against *L. bilineata* carried out in Brazil. The results obtained in the laboratory indicate the high potential of the use of three *B. bassiana* strains against *L. bilineata* as a biocontrol agent.

## 1. Introduction

*Lema bilineata* Germar (Coleoptera: Chrysomelidae) is a defoliating insect that feeds on host plants of the Solanaceae family [1]. It causes considerable economic damage during its larval and adult phases in crops of *Physalis peruviana* (L.) in Chile and Argentina [2]; Italy [3]; and, recently, Brazil [4]. Moreover, this Coleoptera has been reported to damage *Physalis viscosa* (L.) in Australia [5], and tobacco (*Nicotiana tabacum* L.) in Argentina and South Africa [6,7]. Currently, no authorized biological or chemical pesticides for controlling *L. bilineata* are available in Brazil. The damage caused by this coleoptera on *P. peruviana* and the absence of commercial pesticides have led producers and agricultural technicians to seek alternatives [8], which include biological controls [4,9]. In this regard, the search in the wild for natural enemies, such as parasitoids, predators, and entomopathogenic fungi, associated with this coleoptera pest species is crucial for guiding new research on organisms with the potential to be used in conservation biological control strategies or applied biological control programs [4,10,11]. A highly diverse range of entomopathogenic fungi can be naturally present in agroecosystems, acting as regulators of arthropod pest populations [12,13]. However, due to the high biodiversity of these organisms, it is important to know the pathogenicity and degree of virulence of a given fungal strain against a specific pest [9,14].

In 2018, during the collection of *L. bilineata* individuals in *P. peruviana* crops in the Paraná state (Brazil), it was observed that *L. bilineata* adults were infected by *Beauveria bassiana* ((Balsamo-Crivelli) Vuillemin) (Hypocreales: Cordycipitaceae) [4]. Although entomopathogenic fungi have been widely used against a number of insect pests, no data on the efficacy of these fungi against *L. bilineata* are available. In this context, the current study aims to verify the pathogenicity of 15 strains of *B. bassiana* against *L. bilineata* adults and eggs under laboratory conditions. Therefore, this work represents the first step in the development of an improved integrated pest management (IPM) program for this pest.

## 2. Materials and Methods

### 2.1. Isolation and Identification of Entomopathogenic Fungi

Entomopathogenic fungi were isolated from 15 dead *L. bilineata* adults found in a *P. peruviana* crop in a non-commercial field of Campo Largo (25°27′09.8″ S 49°34′06.4″ W), Paraná, Brazil, between September and December 2019. The insects were individualized in sterile microtubes and taken to the laboratory. Subsequently, each insect was placed in a Petri dish with Sabouraud Dextrose Agar (SDA) medium and chloramphenicol and kept at 28 ± 1 °C for five days. Fungal isolates were purified by selecting monosporic colonies, which were then transferred into an SDA medium. Preliminary identification was performed by analyzing micromorphological characteristics under an optical microscope (400x) after 7 and 14 days of growth [15]. The isolates were deposited in the Microbiological Collection of the Paranaense Network—TAXonline (CMRP), Department of Basic Pathology, Federal University of Paraná (Curitiba, Brazil) (http://www.splink.org.br) (accessed on 24 January 2020).

#### 2.1.1. Molecular Identification

The genomic DNA extraction and sequencing reactions were performed according to Vicente et al. [16]. Briefly, DNA was extracted from colonies of about 2 cm^3^ cultivated in SDA by transferring the biological material to a 2 mL microtube containing 300 µL of cetyl trimethyl ammonium bromide (CTAB) and ~80 mg of a silica:celite mixture (2:1). The cells were manually disrupted with a sterile pestle for 5 min. Then, 700 µL of CTAB buffer was added and the microtube was incubated for 60 min at 65 °C. Next, 600 μL of CIA (24:1; chloroform:isoamyl alcohol) was added and centrifuged for 10 min at 12,000 *g*. The supernatant was collected and transferred to a new tube in which 800 µL of ice-cold 100% isopropyl ethanol was added for DNA precipitation for 45 min at −20 °C. The tubes were centrifuged for 15 min at 12,000 *g* and 500 µL of cold 70% ethanol was added to wash the DNA. The pellet was dried at room temperature and resuspended in 100 µL of ultrapure water. DNA purity and integrity were evaluated by spectrophotometry (NanoDrop^®^, Thermo Scientific, Wilmington, DE, USA) and electrophoresis using 1% agarose gel.

The internal transcribed spacer (ITS) of the nuclear rRNA and the elongation factor (ef-1α) gene were chosen for species delimitation. Polymerase Chain Reaction (PCR) amplification was performed using the universal primers ITS1 (5′-TCCGTAGGTGAACCTGCGG-3′; forward) and ITS4 (5′-TCCTCCGCTTATTGATATGC-3′; reverse) for ITS and EF1 (5′-ATGGGTAAGGARGACAAGAC-3′; forward) and EF2 (5′-GGARGTACCAGTSATCATGTT-3′ reverse) for ef-1α. PCR reaction mixes consisted of 1× PCR buffer, 2.0 mM MgCl2, 25 μM deoxynucleoside triphosphates (dNTPs), 10 pmol of each primer, 1 U of Taq DNA polymerase (Ludwig Biotec, Alvorada, Brazil), and 10 ng of gDNA, with a final volume of 12.5 μL. PCR reactions were conducted in an ABI Prism 2720 thermocycler (Applied Biosystems, Foster City, CA, USA), as follows: 95 °C for 4 min; 35 cycles at 95 °C for 45 s, 55 °C for 30 s, and 72 °C for 2 min; and a delay at 72 °C for 7 min. Amplicons of ITS and ef-1α were sequenced with the BigDye Terminator cycle sequencing kit v.3.1 (Applied Biosystems, Foster City, CA, USA) with the same PCR primers and in accordance with the manufacturer’s instructions, as follows: 95 °C for 1 min, followed by 30 cycles at 95 °C for 10 s, 50 °C for 5 s, and 60 °C for 4 s. The sequences were analyzed on an ABI Prism 3700 DNA sequencer (Applied Biosystems, Foster City, CA, USA).

#### 2.1.2. Phylogenetic Analysis

Consensus sequences of the ITS and ef-1α regions were visually inspected using MEGA v.7 [17]. Initially, each gene was analyzed separately by alignments generated in the MAFFT online software [18], with reference strains according to Rehner et al. [19]. Then, the alignment of ITS and ef-1α was concatenated in MAFFT. A phylogenetic analysis was performed in MEGA v.7 using the best evolutionary model that had previously been established through the program, using the maximum likelihood algorithm with 1000 bootstrap replicates. Bootstrap values equal to or greater than 70% were considered statistically significant.

### 2.2. Rearing of Lema bilineata (Coleoptera: Chrysomelidae)

*Lema bilineata* larvae and adults were collected in November 2019 from *P. peruviana* plants in Pinhais, Paraná, Brazil (25°23′30″ S 49°07′30″ W). Larvae were kept until adulthood, and adult specimens were observed under a stereoscopic microscope (Stemi 508, Zeiss; 2.5×) for species confirmation. Insects were reared in the laboratory under controlled conditions (25 ± 1 °C, 70 ± 10% RH, and 14L:10D photoperiod) [4]. The adults were kept in 120 mL plastic containers with small orifices for gas exchange. For feeding and substrate laying, *P. peruviana* leaves were kept inside the containers with their petioles immersed in 2 mL conical bottomed polypropylene microtubes filled with water to prevent turgor loss. Leaves with eggs were transferred to Petri dishes (9 cm in diameter), with their petioles also immersed in 2 mL conical microtubes with water.

The insects were fed ad libitum with *P. peruviana* leaves, which were replaced on a daily basis. When the larvae reached the pre-pupa stage, they were transferred into Petri dishes containing moistened filter paper, where they progressed to the pupa stage. They remained in the Petri dishes until reaching adulthood, when they were transferred to new plastic containers.

### 2.3. Pathogenicity Assays

#### 2.3.1. Inoculum Preparation

All *Beauveria* isolates were grown separately in Petri dishes (9 cm in diameter) containing SDA medium and incubated at 28 °C for 14 days. Then, conidia were scraped from the medium using a sterile spatula and transferred to glass vials containing 15 mL of 0.85% saline solution (NaCl containing 0.01% Tween^®^ 80). Conidia suspensions were vortexed for 2 min, filtered, and transferred to test tubes (30 mL). New conidial suspensions were prepared for each bioassay and used immediately after preparation. The concentration of conidia was adjusted to 10^8^ spores mL^−1^ using a hemocytometer. Conidial viability was determined before the suspension preparation by observing whether 100 spores could be seen under an optical microscope (400× magnification) after 12 h of growth. Conidia with a germ tube were considered viable.

#### 2.3.2. Bioassay: Effects of Fungal Isolates on Adults of *Lema bilineata* (Coleoptera: Chrysomelidae)

*Physalis peruviana* leaves were disinfected superficially and placed in 120 mL plastic containers (one leaf per container) [4]. The experimental design was completely randomized. Each treatment (fungal isolate) was conducted with five replications. Two *L. bilineata* adults were transferred to each container, which was considered a replication, for a total of 150 individuals tested (10 per treatment). A Sagyma SW776 airbrush (10 lb pol^−1^) was used to spray 1 mL of conidial suspension at a concentration of 2.17 × 10^8^ spores mL^−1^ and 0.1% Tween^®^80 over the *L. bilineata* individuals. The control treatment received 1 mL of sterile distilled water with 0.1% (*v*/*v*) Tween^®^80. After spraying, the plastic containers were kept under controlled conditions (25 ± 2 °C, 60 ± 10% RH, and 12:12 h (L:D) photoperiod). Mortality was assessed after a seven-day period. Moribund individuals or those that did not respond to touch with a paintbrush were considered dead. To confirm the cause of death, these specimens had their body surfaces disinfected in 0.1% sodium hypochlorite solution [20] and were then transferred to sterile microtubes until fungal extrusion to check for postmortem sporulation. The bioassay was carried out for three weeks (with 5 different treatments weekly).

#### 2.3.3. Bioassay: Effects of Fungal Isolates on the Viability of *Lema bilineata* (Coleoptera: Chrysomelidae) Eggs

In this case, the three *B. bassiana* isolates that proved to be more virulent against *L. bilineata* adults were tested on eggs. A total of 675 *L. bilineata* eggs were used for this bioassay, with each replicate consisting of a *Physalis peruviana* leaf with 25 *L. bilineata* eggs. The methodology was similar to that explained for the former bioassay. *Physalis* peruviana were disinfected superficially and were placed in 120 mL plastic containers (one leaf per container) [4]. The experimental design was completely randomized. Each treatment (fungal isolate) was conducted with nine replications. A Sagyma SW776 airbrush (10 lb pol^−1^) was used to spray 1 mL of conidial suspension at a concentration of 2.04 × 10^8^ spores mL^−1^ and 0.1% Tween^®^80 over the *L. bilineata* eggs. The control treatment received 1 mL of sterile distilled water with 0.1% (*v*/*v*) Tween^®^80. After spraying, plastic containers were kept under controlled conditions (25 ± 2 °C, 60 ± 10% RH, and 12:12 h (L:D) photoperiod). Mortality was assessed after seven days. Dry and unhatched eggs were considered dead. The bioassay was carried out for three weeks.

### 2.4. Statistical Analysis

Mean mortality rates (%) were used to calculate fungal efficiency and the treatments were compared using Tukey’s test (*p* < 0.05). Mortality rates were corrected using Abbott’s equation [21]. Data were checked for normality and homocedasticity.

## 3. Results and Discussion

### 3.1. Isolation and Identification of Entomopathogenic Fungi

In the end, 15 isolates were morphologically identified as belonging to the genus *Beauveria* and the molecular analysis confirmed that all isolates were *Beauveria bassiana* (Table 1).

**Figure 1:**
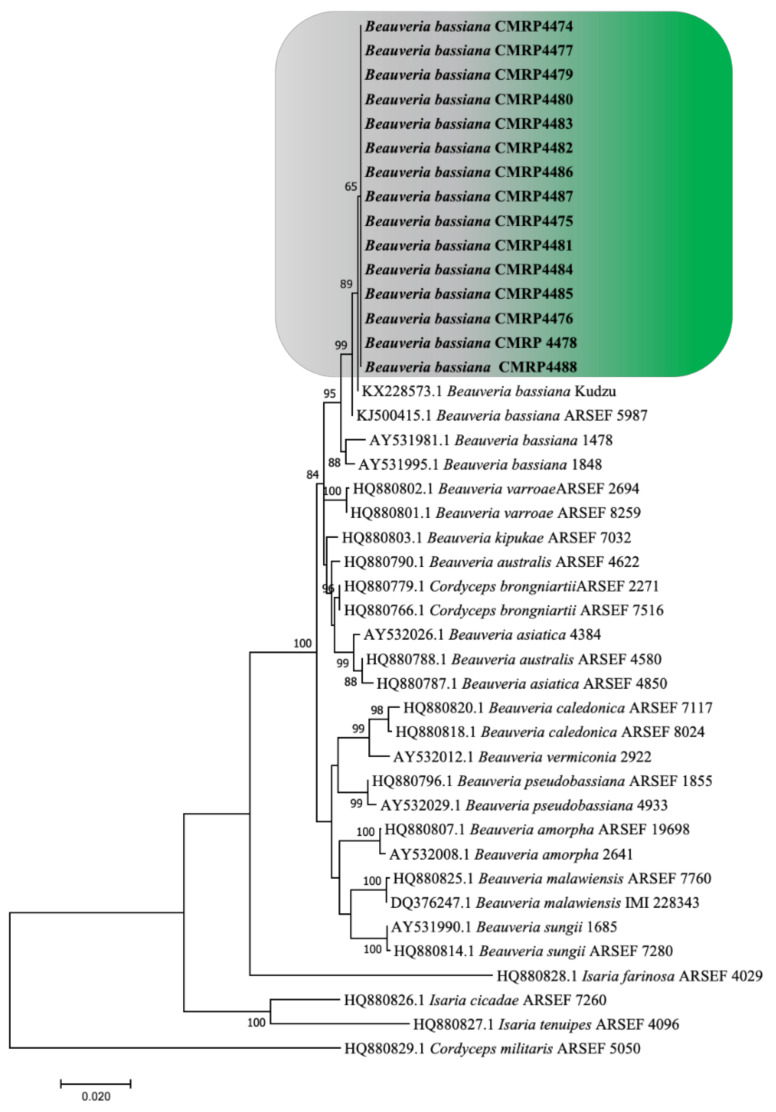
Maximum likelihood phylogenetic tree of the *Beauveria* species recovered in the MEGA 7.0 software based on the concatenated alignment of the transcribed internal spacer (ITS) and the elongation factor 1-alpha (ef-1α) gene, using the Tamura-Nei with gamma invariable sites (TN93 + G + I) model. Bootstrap support was calculated from 1000 replicates. *Cordyceps militaris* ARSEF 5050 was used as an outgroup. The highlighted CMRP isolates form a separate group from the other *Beauveria* strains included in the study.

The phylogenetic tree (Figure 1) showed that the fungi isolated in the current study were in close proximity (95%) with the *B. bassiana* strains reported by Rehner et al. [19]. In addition, the isolated strains formed a separate clade from the other sequences in the group, although bootstrapping slightly supported this clade (65%). In the end, the 15 isolated strains were classified as *B. bassiana* (Figure 1). This fungal species has entomopathogenic characteristics and the potential to be used as a biological agent for controlling insect pest populations of different crops, as reported in several studies [9,10,11,12,13]. Further investment in biological control with entomopathogenic fungi could contribute to sustainable crop production either as a stand-alone strategy or in support of other biological and IPM strategies [22].

### 3.2. Pathogenicity Tests against Lema bilineata

#### 3.2.1. Pathogenicity Test in *Lema bilineata* Adults

The 15 isolates (identified as CMRP4474–CMRP4488, as indicated in Figure 1) were used in the pathogenicity test with *L. bilineata* adults. All fungal isolates presented germ tube growth after 12 h in an SDA medium and were considered viable with over 90% germination. This first screening for selecting the most efficient entomopathogen against *L. bilineata* is a crucial step for developing biocontrol strategies against this pest. With germination rates above 90% and a short time of action, the tested isolates were promising prospects for the control of *L. bilineata*.

In the current study, the 15 isolates tested showed efficacy against *L. bilineata* adults, with varying mortality rates between 20% and 80% (Table 2). A statistical difference (*p* < 0.05) was detected between the CMRP4480, CMRP4487, and CMRP4488 strains and the other isolates. These three isolated strains showed the highest pathogenicity (causing 80% mortality) against *L. bilineata* adults (Table 2). In contrast, CMRP4474, CMRP4478, and CMRP4484 showed the lowest pathogenicity against *L. bilineata* adults (Table 2). The observed variability in mortality rates is expected among isolates that are tested for the first time [12]. The life cycle of entomopathogenic fungi on the insect begins with spore germination and cuticle penetration, followed by hyphae proliferation, which ends up killing the hosts. This cycle is repeated countless times, with the production of infective spores that can penetrate immediately into the cuticle of other individuals to repeat the cycle [23]; this process may promote the medium-term maintenance of the fungal presence in the crop.

**Figure 2:**
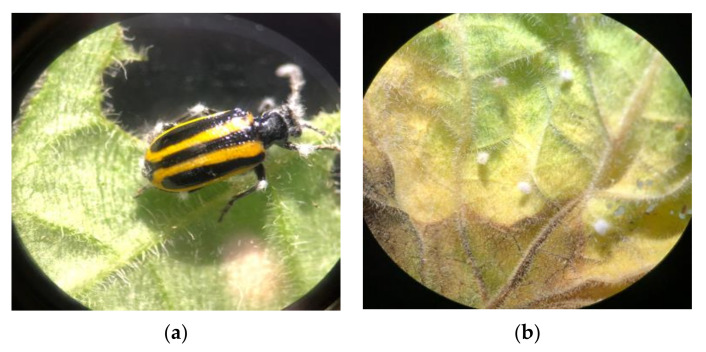
Photographs of *Lema bilineata* showing signs of fungal extrusion: (**a**) adult with first signs of fungal extrusion; (**b**) eggs with fungal extrusion.

A total of 100 insects died in the current experiment. They were individualized in sterile microtubes, and 76 individuals showed fungal extrusion, confirming the isolated fungal strains as the causal agents of death (Figure 2a).

#### 3.2.2. Pathogenicity Test in *Lema bilineata* Eggs

When performing the former bioassay using *L. bilineata* adults, fungi signals were observed in eggs a few days after those eggs were laid by *L. bilineata* females sprayed with *B. bassiana* conidial suspensions. According to these observations, *B. bassiana* isolates CMRP4480, CMRP4487, and CMRP4488, which were the most virulent in the bioassay with adult individuals (Table 2), were tested for their pathogenicity against *L. bilineata* eggs. We observed that between 58.7% and 66.7% of eggs were unhatched after seven days (Table 3). In this case, CMRP4480 was the most virulent isolate, being significantly different (*p* < 0.05) from the rest of the isolates (Table 3).

All unhatched eggs that underwent spraying came to be considered dead, since the non-hatching rate in the control groups was only 8.8%. Fungal sporulation was detected in most eggs that did not hatch (Figure 2b).

## 4. Conclusions

This is the first study about entomopathogenic fungi isolated from *P. peruviana* crops to be tested against *L. bilineata*. All 15 fungal isolates tested had different levels of pathogenicity. The CMRP4480, CMRP4487, and CMRP4488 isolates induced the highest mortality rates in *L. bilineata* adults and unhatched eggs. Our findings in the laboratory showed that *B. bassiana* isolates, as biological control agents with pathogenic potential against *L. bilineata*, are a promising alternative to the traditional chemical insecticides that are currently employed for this task. However, these results must be expanded with experiments in open fields in order to evaluate entomopathogenic fungi action in uncontrolled conditions as a step forward in the development of better IPM approaches and their use as a biopesticide. The data presented here could help to improve the initial development of IPM strategies for *P. peruviana* crops, since no synthetic insecticides are available for the specific control of *L. bilineata* in Brazil.

## Figures and Tables

**Table 1:** Fungal strains, identified as *Beauveria bassiana*, isolated from *Lema bilineata* (Coleoptera: Chrysomelidae) adults collected in Campo Largo, Paraná, Brazil, and their GENBANK accession numbers.

Isolate	Identification		GENBANK Accesion Number	
	Morphological	Molecular	ITS	ef-1α
CMRP4474/L1	*Beauveria* sp.	*B. bassiana*	MZ567032	
CMRP4475/L2			MZ567033	
CMRP4476/L3			MZ567034	
CMRP4477/L4			MZ567035	
CMRP4478/L5			MZ567036	
CMRP4479/L6			MZ567037	
CMRP4480/L7			MZ567038	MZ574443
CMRP4481/L8			MZ567039	
CMRP4482/L9			MZ567040	
CMRP4483/L10			MZ567041	
CMRP4484/L11			MZ567042	
CMRP4485/L12			MZ567043	
CMRP4486/L13			MZ567044	
CMRP4487/L14			MZ567045	MZ574442
CMRP4488/L15			MZ567046	

**Table 2:** Total number of *Lema bilineata* Germar (Coleoptera: Chrysomelidae) adults dead and adjusted mortality rate after inoculation with conidial suspensions (10^8^ conidia mL^−1^) of isolated *Beauveria bassiana* strains.

Isolate	Mortality	
	Total Number of Dead *Lema bilineata* Adults	Rate (%)
Control	3	0 a
CMRP4474	4	20 a
CMRP4475	8	60 a
CMRP4476	4	40 a
CMRP4477	6	40 a
CMRP4478	6	20 a
CMRP4479	6	60 a
CMRP4480	10	80 b
CMRP4481	8	60 a
CMRP4482	6	40 a
CMRP4483	8	60 a
CMRP4484	4	20 a
CMRP4485	4	40 a
CMRP4486	6	60 a
CMRP4487	10	80 b
CMRP4488	10	80 b

Note: Different letters in the column indicate significant differences among isolated fungal strains according to the Tukey test (*p* < 0.05).

**Table 3:** Mortality rates of *Lema bilineata* Germar (Coleoptera: Chrysomelidae) eggs after inoculation with conidial suspensions (10^8^ conidia mL^−1^) of three isolated *Beauveria bassiana* strains. Data are means ± standard deviations.

Isolate	Rate (%)
Control	6.7 ± 1.8 a
CMRP4480	66.7 ± 1.6 c
CMRP4487	60.0 ± 2.0 b
CMRP4488	58.7 ± 2.4 b

Note: Different letters in the column indicate significant differences among isolated fungal strains according to the Tukey test (*p* < 0.05).
